# Predicting Protein Dynamics and Allostery Using Multi-Protein Atomic Distance Constraints

**DOI:** 10.1016/j.str.2017.01.008

**Published:** 2017-03-07

**Authors:** Joe G. Greener, Ioannis Filippis, Michael J.E. Sternberg

**Affiliations:** 1 Centre for Integrative Systems Biology and Bioinformatics, Department of Life Sciences, Imperial College London, London SW7 2AZ, UK

**Keywords:** distance geometry, ensemble, allostery, protein dynamics, T4-lysozyme, cyclin-dependent kinase 2, catabolite activator protein, stochastic proximity embedding

## Abstract

The related concepts of protein dynamics, conformational ensembles and allostery are often difficult to study with molecular dynamics (MD) due to the timescales involved. We present ExProSE (Exploration of Protein Structural Ensembles), a distance geometry-based method that generates an ensemble of protein structures from two input structures. ExProSE provides a unified framework for the exploration of protein structure and dynamics in a fast and accessible way. Using a dataset of apo/holo pairs it is shown that existing coarse-grained methods often cannot span large conformational changes. For T4-lysozyme, ExProSE is able to generate ensembles that are more native-like than tCONCOORD and NMSim, and comparable with targeted MD. By adding additional constraints representing potential modulators, ExProSE can predict allosteric sites. ExProSE ranks an allosteric pocket first or second for 27 out of 58 allosteric proteins, which is similar and complementary to existing methods. The ExProSE source code is freely available.

## Highlights

• ExProSE uses distance geometry to generate protein ensembles from two structures
• Generated ensembles are native-like spanning conformational changes
• Allosteric sites are predicted with performance complementary to existing methods
• ExProSE is open-source, accessible, and effective without user parameter selection

## Graphical Abstract

**Figure:**
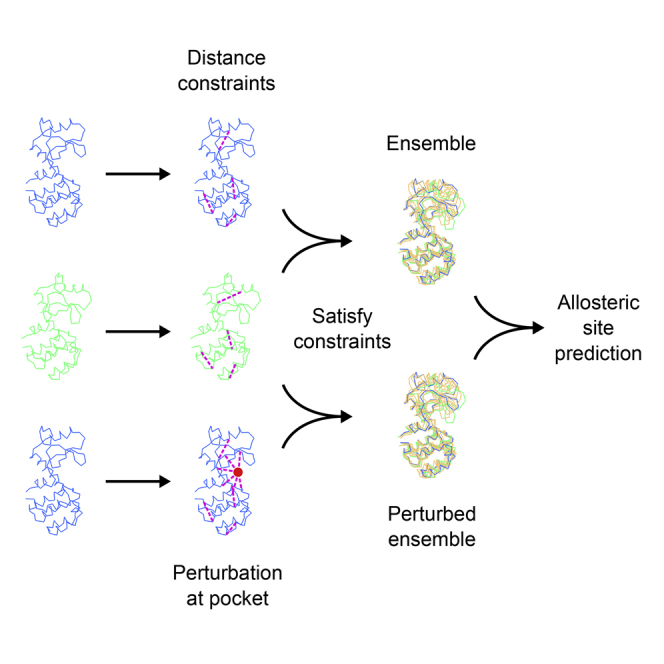

## Teaser

Greener et al. present a computational method that generates an ensemble of protein structures from two input structures. ExProSE gives native-like ensembles that span conformational space, and can predict allosteric sites. The method is a quick and accessible way to explore protein structure and dynamics.

## Introduction

Proteins move on a variety of timescales, encompassing motions from the vibration of a single bond to the collective movement of whole domains (Henzler-Wildman and Kern, 2007, Wei et al., 2016). X-ray crystallography provides a static view of the structure of proteins. However, when only static structures are available the dynamic processes crucial to protein function (Henzler-Wildman et al., 2007) are difficult to elucidate. Experimental techniques to explore the dynamics of proteins, such as nuclear magnetic resonance (NMR), are sophisticated and time-consuming. Molecular dynamics (MD) is a widespread computational method for predicting protein motions and generating ensembles of protein structures. It is effective at modeling motions up to the timescale of nanoseconds. However, the computational cost of modeling proteins on the scale of microseconds or milliseconds means that MD is not suitable for larger-scale transitions. Advanced MD methods such as targeted or accelerated MD can overcome this sampling problem (Maximova et al., 2016), but these methods are not yet routinely applicable due to the parameterization required for each protein.

Various non-MD methods have been used to generate ensembles of protein structures from a crystal input structure, and hence explore protein dynamics. These ensembles have uses in flexible ligand docking (Totrov and Abagyan, 2008), generating poses for protein-protein docking (Mustard and Ritchie, 2005), predicting structures on trajectories between two crystal structures (Weiss and Levitt, 2009), and predicting flexible regions in proteins (Ahmed et al., 2011).

CONCOORD (de Groot et al., 1997, de Groot et al., 1999) is a distance geometry method to generate structures from an input structure, and consists of a two-step process. First, the different types of chemical interactions in the input structure, e.g., hydrogen bonding and hydrophobic interactions, are converted to distance constraints with a given tolerance. Next, an iterative minimization procedure is performed to move a set of randomly placed coordinates such that most distance constraints are satisfied. This generates a protein structure in a manner similar to the way a structure is produced from NMR constraints. The process is repeated to obtain an ensemble of structures. tCONCOORD extends CONCOORD and gives better sampling of proteins with large conformational changes by predicting hydrogen bonds in the structure that are liable to break (Seeliger et al., 2007).

Normal mode analysis (NMA) can also be used to generate conformations of proteins, usually by modeling the protein along the relevant vibrations. The NMSim web server (Kruger et al., 2012, Ahmed et al., 2011) finds flexible and rigid protein regions using the graph theoretical approach FIRST (Jacobs et al., 2001), then generates conformations along low-frequency normal modes. The generated structures are iteratively corrected to produce valid stereochemistry.

Modeling conformational transitions is essential in understanding biological processes such as allostery, whereby an effector at a site distant from the active site causes a change in structure or dynamics that leads to a functional change in the protein (Nussinov and Tsai, 2013). Allostery can arise from non-covalent interactions (e.g., drug binding), covalent interactions (e.g., phosphorylation) and light absorption. This intrinsic property of proteins (Gunasekaran et al., 2004) is important in processes such as cellular signaling and disease, although most allosteric mechanisms remain an enigma and a universal mechanism has not been found (Nussinov and Tsai, 2013).

The discovery of new allosteric modulators is of pressing concern, due to their considerable potential as therapeutics (Lamba and Ghosh, 2012). Allosteric modulators have been elucidated for targets as diverse as the γ-aminobutyric acid receptor, hepatitis C virus polymerase, and RNA. Allosteric modulator discovery by virtual screening is an exciting prospect furthered by the elucidation of previously unknown allosteric sites found on solved protein structures (Panjkovich and Daura, 2010). There is an increasing number of entries in the AlloSteric Database (ASD) (Shen et al., 2016), which currently contains more than 1,400 proteins. This shows that a large variety of proteins have allosteric character and implies that many proteins have allosteric character yet to be discovered. However, discovery of allosteric drugs presents challenges beyond those encountered in orthosteric drug discovery. Whether the drug will activate or inhibit the protein is difficult to predict, and in many cases the location of allosteric sites is unknown. Existing approaches for allosteric site prediction include using changes in flexibility on ligand binding (Mitternacht and Berezovsky, 2011, Panjkovich and Daura, 2012, Greener and Sternberg, 2015), machine learning on pocket features (Huang et al., 2013, Cimermancic et al., 2016) and structural conservation (Panjkovich and Daura, 2010).

Allostery can be thought of as a property of the ensemble of available protein structures (Motlagh et al., 2014). A perturbation at any site in the structure leads to a shift in the occupancy of states by the population. The conformational selection paradigm suggests that all states available to the protein pre-exist, but certain states (e.g., an allosteric inactive state) are only significantly populated when the allosteric modulator is present. If a method can model the structural ensemble in such a way that the effect of modulators can be predicted, sites with allosteric character can be found.

Here we present a novel distance geometry-based method, named ExProSE (Exploration of Protein Structural Ensembles), for protein ensemble generation and allosteric site prediction. By using distance constraints from two crystal structures, ExProSE produces ensembles of protein structures that sample biologically relevant conformations. The ensemble differs from an ensemble arising from MD. The structures are not a snapshot in time on a trajectory; instead, each structure is generated independently. We show that ExProSE provides better coverage of the conformational space than existing methods. Allosteric sites on a set of proteins are predicted by examining the effect of potential modulators on the population distribution of the ensemble. To our knowledge, this is the first study to integrate available structural data into a general framework that allows exploration of protein dynamics and allostery, and that provides models for further studies such as ligand docking.

## Results

ExProSE is able to (1) generate ensembles of protein structures from two input structures and (2) predict allosteric pockets on proteins. First, it is shown using a dataset of structural pairs that two widely used methods for generating ensembles cannot span large conformational changes. The ability of ExProSE to produce native-like ensembles is exemplified with T4-lysozyme. ExProSE ensembles can be perturbed to reveal the location of allosteric sites, as demonstrated on cyclin-dependent kinase 2 (CDK2). The performance of ExProSE in predicting allosteric sites is assessed on a dataset of 58 known allosteric proteins. Finally, a well-studied example of dynamic allostery is examined.

### Ensemble Generation

#### Apo/Holo Dataset

To examine the ability of existing non-MD methods to generate ensembles that span conformational changes, we used a dataset of apo (no modulator) and holo (modulator bound) structures (Atilgan et al., 2010). The proteins have a root-mean-square deviation (RMSD) between apo and holo structures ranging from 2 to 19 Å, and represent a variety of domain, subdomain, and subunit motions. tCONCOORD (Seeliger et al., 2007) and NMSim (Kruger et al., 2012) both seek to model conformational changes such as those in the dataset. Default parameters were used to produce 250 structures for each protein from tCONCOORD and NMSim. The lowest RMSD of the structures in an ensemble to a particular crystal structure was taken as a measure of how close the ensemble came to exploring the conformational space of that crystal structure. This can be seen in Table 1.

When the apo structure is used as input, structures similar to the apo structure are generated by both methods. The median lowest RMSD to the apo crystal is 1.44 Å for tCONCOORD and 0.71 Å for NMSim. However, structures similar to the holo crystal are not sampled. The median lowest RMSD to the holo crystal is 4.15 Å for tCONCOORD and 4.68 Å for NMSim. In a similar manner, when the holo structure is used as input to tCONCOORD and NMSim, the ensembles sample the holo structure but not the apo structure.

ExProSE, as expected because it uses both the apo and holo crystals as input, is able to generate structures close to both crystals (Table 2). For 11 out of the 12 proteins ExProSE can generate a structure closer to the holo crystal than the other methods, where the other methods use the apo structure as input. For the opposite case, compared with the apo crystal, ExProSE also generates a closer structure for 11 out of 12 proteins. Hence ExProSE is useful for generating ensembles when two or more structures are available.

PROCHECK checks the stereochemical quality of protein structures (Laskowski et al., 1993). The PROCHECK overall G factor is a log-odds score based on the observed distributions of various stereochemical parameters in reference proteins. A lower overall G factor represents a low-probability conformation and indicates a less stereochemically valid structure. Ideally, scores should be above −0.5, and values below −1.0 may need investigation (Esposito et al., 2006). The median PROCHECK overall G factor across all generated structures is −0.99 for ExProSE, indicating that PROCHECK produces structures that are generally acceptable. The values for NMSim and tCONCOORD are −0.32 and −1.83, respectively, indicating that NMSim produces good-quality structures and tCONCOORD produces structures with poor stereochemical quality. The stereochemistry of generated structures can be improved by energy minimization (see below).

#### T4-Lysozyme

Here, we demonstrate that ExProSE can generate structures close to crystals not used as input. Lysozymes damage bacterial cell walls by catalyzing the hydrolysis of peptidoglycans. Bacteriophage T4-lysozyme is a suitable protein for analyzing conformational variability, as there are many crystal structures available and MD simulations of the protein have shown that simulations up to 200 ns do not reliably reach both the open and closed conformations (Seeliger et al., 2007). The pairwise RMSDs of the crystals range from 0.64 to 4.25 Å.

**Figure 1:**
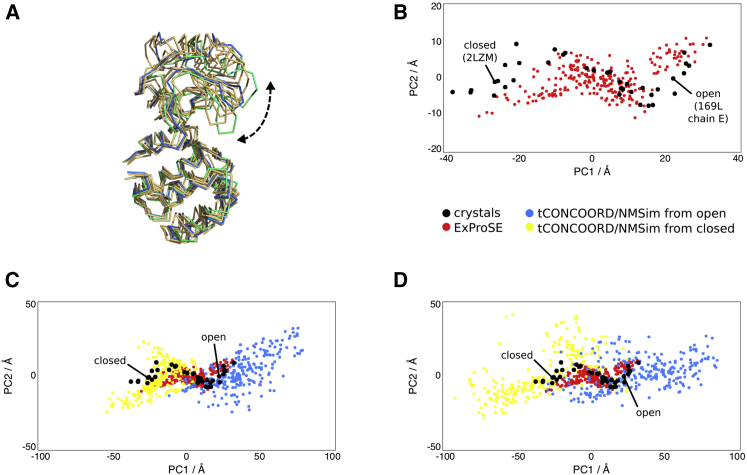
T4-Lysozyme Ensembles (A) Four structures generated from ExProSE using the open (PDB: 169L, chain E) and closed (PDB: 2LZM) conformations as input are shown in orange. The crystal structures of the open and closed conformations are shown in blue and green, respectively, for reference. The arrow shows the opening motion caused by the breaking of a hydrogen bond between Arg137 and Glu22. (B) Projections of the 38 crystal structures used in a prior study (de Groot et al., 1998) onto the first (x axis) and second (y axis) PCs of the PCA of the crystal structures, which account for 70% and 12% of the motion, respectively (black dots). Projections from the ensembles generated with ExProSE are also shown (red dots). (C) Projections of two tCONCOORD ensembles on the same plot as (B). An ensemble using the open structure as input (blue dots) and an ensemble using the closed structure as input (yellow dots) are shown. (D) Projections of two NMSim ensembles with parameters for large-scale motions on the same plot as (B). An ensemble using the open structure as input (blue dots) and an ensemble using the closed structure as input (yellow dots) are shown. See also Figure S1 and Table S1.

An ensemble was generated using ExProSE from the open (PDB: 169L, chain E) and closed (PDB: 2LZM) conformations. Four random structures from this ensemble are shown in comparison with the open and closed crystal structures in Figure 1A. Principal components analysis (PCA) can be carried out on an ensemble of structures to find the orthogonal motions that describe the variation in the ensemble. Figure 1B shows the projections of the generated ensemble and the 38 crystal structures used in a prior study (de Groot et al., 1998) onto the first and second principal components (PCs), which account for 70% and 12% of the motion, respectively. The dominant first eigenvector corresponds to opening and closing of the structure. It can be seen that the method is able to sample conformations corresponding to experimentally observed structures, as the ensembles largely overlap.

Ensembles produced by tCONCOORD starting from the open and closed structures separately are shown in Figure 1C. As demonstrated previously on other proteins, the ensemble generated from the open structure cannot reach all the way to the closed structure, and vice versa. The tCONCOORD ensembles also sample structures not found in the ensemble of crystal structures, particularly when using the open conformation as input. This tendency of tCONCOORD to produce ensembles with too much structural variability was also noted by the authors (Seeliger and de Groot, 2009).

Ensembles produced by NMSim starting from the open and closed structures separately are shown in Figure 1D. In this case, the ensemble generated from the open and closed structures can largely span the conformational space. Similar to tCONCOORD, regions not explored by the crystals are sampled by NMSim. For example, there is one model in the ensemble generated from the open structure that has an RMSD of 7.38 Å to the nearest crystal structure.

Alternative parameters were also used for tCONCOORD and NMSim to discern how the ensembles varied (Figure S1). For tCONCOORD, decreasing the upper bound for long-range constraints and/or turning off close pairs as constraints had little effect on the distribution of the ensembles. For NMSim, using the parameters for small-scale motions led to ensembles that could not span the conformational space. In each case the default parameters gave similar or better coverage of the conformational space of the crystals by visual inspection, and were hence used for the analysis described below.

**Figure 2:**
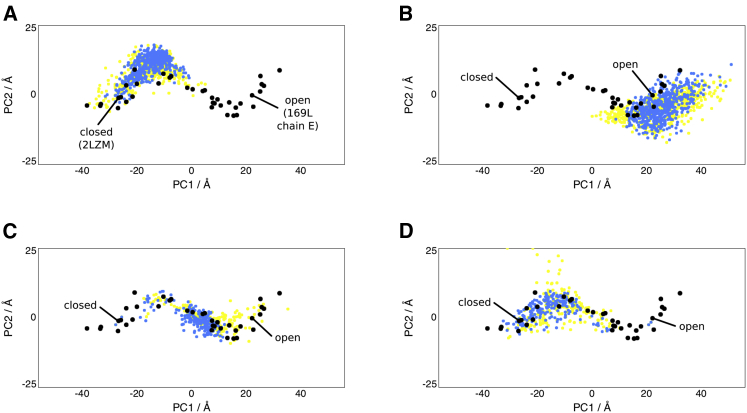
MD T4-Lysozyme Ensembles Projections of two repeats of a particular MD run onto the PCA of the crystal structures are shown (blue and yellow dots), with snapshots taken every 100 ps. Similarly to Figure 1, in each graph the projections of the crystals are also shown (black dots). (A) 50-ns MD runs starting from the closed structure (PDB: 2LZM). (B) 50-ns MD runs starting from the open structure (PDB: 169L). (C) 20-ns targeted MD runs starting from the closed structure and targeting the open structure. (D) 20-ns targeted MD runs starting from the open structure and targeting the closed structure.

T4-lysozyme was also studied with MD. MD runs of 50 ns starting from the closed conformation were not able to reach the open conformation and vice versa (Figures 2A and 2B). Targeted MD runs starting from the closed conformation and targeting the open conformation (and vice versa) were also carried out. In targeted MD the atoms are guided to a target structure with the use of a steering force that seeks to minimize the RMSD of the structure to the target structure. These ensembles can be seen in Figures 2C and 2D, and are generally able to cross conformational space over the course of around 20 ns. However, beyond this time they show unpredictable behavior and can deviate from the experimental structures. Retaining only the structures up to 20 ns, as in Figures 2C and 2D, gives ensembles that largely overlap with the experimental structures.

**Figure 3:**
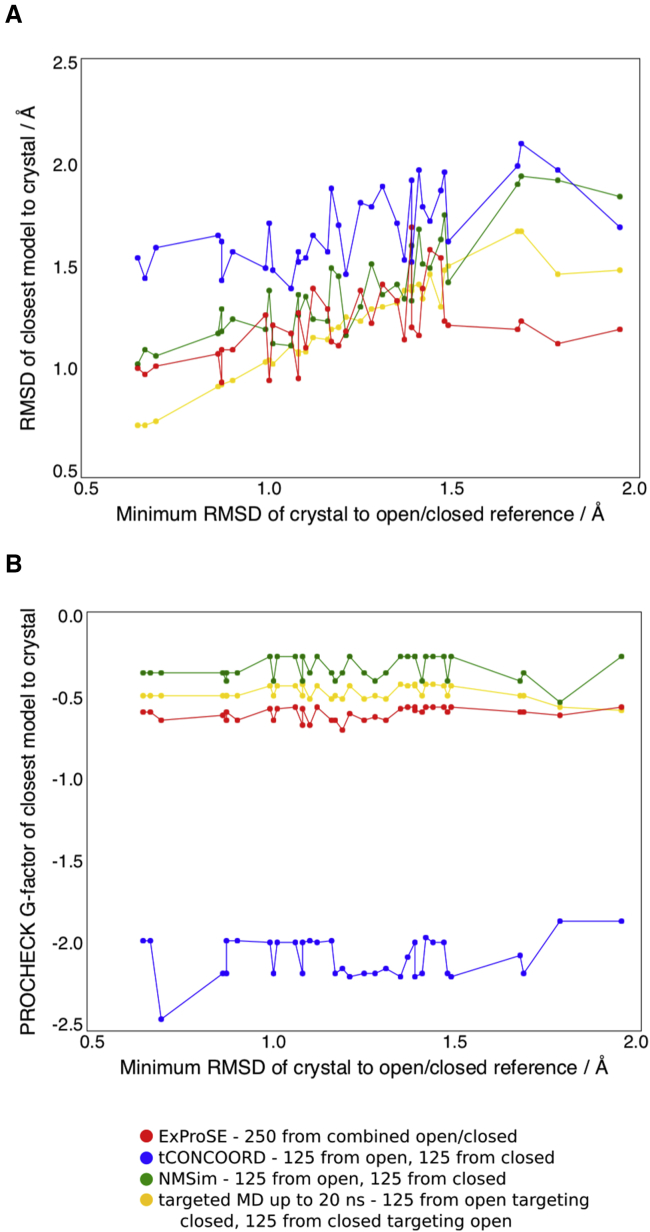
Closest Models from Each Ensemble to T4-Lysozyme Crystal Structures (A) The RMSD of the closest model from each generated ensemble to the crystal structures. The crystal structures are sorted by the lower of the two RMSD values to the open and closed crystals used as input. The crystals used as inputs are omitted from the graph. (B) PROCHECK overall G factors of the closest model from each generated ensemble to the crystal structures. The crystal structures are sorted as in (A).

By combining tCONCOORD, NMSim, and targeted MD ensembles generated using the open and closed structures as input, a fair comparison with ExProSE can be made. A generated ensemble should ideally contain models close to all the crystal structures. The degree to which this occurs for ExProSE ensembles, and combined ensembles for tCONCOORD, NMSim, and targeted MD up to 20 ns, is shown in Figure 3A. It can be seen that ExProSE is able to generate structures close to all crystals, with all crystals having an RMSD of 1.7 Å or less to a generated structure. For 26 out of 38 crystals ExProSE generates a model closer to the crystal than NMSim, and generates a closer model than tCONCOORD in all cases. For 15 out of 38 crystals ExProSE generates a model closer to the crystal than targeted MD. However, this is the case for 14 out of the 27 structures that have an RMSD of more than 1.0 Å to both the open and closed reference structures. Of these 27, ExProSE performs better for all of the four structures that have an RMSD of more than 1.5 Å. Hence, ExProSE is able to generate better models than the other methods for crystals which are far from either input structure, as seen on the right side of Figure 3A. The PROCHECK overall G factor of the closest models for each method is shown in Figure 3B. ExProSE is able to produce models of acceptable quality close to all the crystals, even for those further from the input structures.

To determine whether the stereochemical quality of generated structures could be improved, we carried out energy minimization on all structures. For all methods, energy minimization improved median PROCHECK overall G factors. Across the ensembles the median values increased from the range [−2.23, −0.45] to the range [−0.31, −0.17] (Table S1). This shows that stereochemical problems in generated structures can in general be improved by energy minimization, which is important if using generated structures for docking studies.

By using two input structures rather than one, ExProSE is able to produce models of acceptable quality close to that of other crystal structures. It can explore conformational space better than methods that use a single structure as input.

### Ensemble Perturbation for CDK2

Here, we demonstrate that ExProSE ensembles can be perturbed to reveal modulating sites. CDK2 is a protein kinase essential for the G_1_/S phase transition in the cell cycle (Peyressatre et al., 2015). It associates with, and is regulated by, cyclins. It has been a major target of drug discovery efforts due to its essential role. An ExProSE ensemble was generated using the apo native structure (PDB: 1HCL) and the holo structure bound to two ANS molecules in an allosteric site (PDB: 3PXF). The ANS-bound structure is inactive, as ANS binding causes a conformational shift in the C helix that prevents cyclin binding (Betzi et al., 2011). A further screening study has found potential modulators for the ANS binding site (Rastelli et al., 2014).

**Figure 4:**
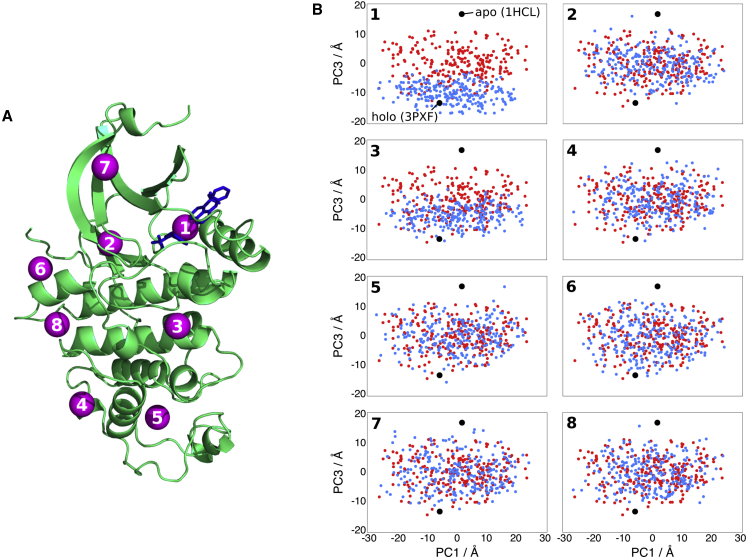
CDK2 Pockets and Projections of Ensembles (A) CDK2 in its holo conformation bound to two ANS molecules in the allosteric site (PDB: 3PXF). CDK2 is shown as a green cartoon with the two bound ANS shown as blue sticks. Pocket centers predicted by LIGSITE^*cs*^ are shown as purple spheres. The pockets are numbered by descending volume. Pocket 1 represents the ANS allosteric pocket. Pocket 2 represents the ATP binding pocket. (B) Structures generated using ExProSE, with input structures the apo and holo structures (PDB: 1HCL and 3PXF, respectively), are shown as red dots. The axes are projections onto the first (x axis) and third (y axis) PCs of the ExProSE ensemble, which account for 35% and 8% of the motion, respectively. The blue dots represent the structures in the ensemble with perturbation at pocket centers 1–8 from (A).

Figure 4A shows the pockets predicted by LIGSITE^*cs*^ (Huang and Schroeder, 2006) on CDK2 bound to two ANS molecules. The ensemble perturbation procedure was carried out at each of the eight pocket centers as described in Experimental Procedures. In brief, additional constraints are added representing a modulator bound in the selected pocket. Projections of the structures of the unperturbed ensemble and the structures of the ensemble with perturbation at the pocket center are shown in Figure 4B, one graph per pocket center. The third PC was chosen for visualization instead of the second as it represents the inactivating motion of the C helix, whereas the second PC represents a rotation in the region of the protein considered to be functionally less important, the C lobe.

Site 1 in Figures 4A and 4B is the ANS allosteric pocket. Simulating a modulator there shifts the ensemble toward the inactive state, agreeing with previous experimental data (Betzi et al., 2011). Site 2 is the ATP binding site, where there is no change in the ensemble upon simulating a modulator there. This is encouraging, as ATP binding does not cause structural changes that lead to cyclin dissociation. Site 3 is found in a pocket near the activation segment. A shift in the ensemble toward the inactive state is seen on perturbation at this site. In fact, this site is close to a potential allosteric site suggested in another computational study (Pitt et al., 2014) and is part of the region associated with cyclin binding. This indicates that the site could potentially be an allosteric site, although further effort would be required to determine whether it is druggable. Simulating modulators at sites 4–8 does not shift the ensemble, suggesting that binding at these sites is unable to cause an allosteric effect. No allosteric modulators have been reported experimentally for these sites.

### Allosteric Site Prediction

Systematic methods to predict allosteric sites on proteins are necessary to utilize the potential advantages of allosteric drugs. A diverse dataset of 58 apo/holo pairs representing the unbound protein and the protein bound to a known allosteric modulator was assembled from the ASD (Shen et al., 2016). This dataset showed a large range in protein size (153–955 residues) and included a variety of proteins including transcriptional regulators, transporters, and protein kinases.

LIGSITE^*cs*^ was used to predict pockets on the holo crystal structures and ExProSE was used to generate a perturbed ensemble for each pocket center, as described in Experimental Procedures. These perturbed ensembles were used to rank the pockets in terms of predicted allosteric effect. In this study a correct prediction for a protein indicates that an allosteric pocket was ranked first or second. This criterion was chosen as a measure of success because typically the top few pockets predicted by a method would be examined and studied further.

The ability of ExProSE to predict allosteric pockets on the dataset is compared with existing allosteric prediction methods, which are run with the holo crystal structures as input. This was found to give better results for the existing methods than using the apo crystals. PARS (Panjkovich and Daura, 2014) uses NMA with and without a predicted modulator to predict changes in flexibility. STRESS (Clarke et al., 2016) is an implementation of the earlier binding leverage algorithm (Mitternacht and Berezovsky, 2011), which models how perturbations due to binding couple to the motions of the protein as expressed by low-frequency normal modes. AlloPred (Greener and Sternberg, 2015) uses perturbation of normal modes and pocket features in a machine-learning approach to predict allosteric pockets. It should be noted that different criteria are used to define an allosteric pocket for each method, due to the nature of their output (see Experimental Procedures). For 27 of 58 proteins ExProSE ranked an allosteric pocket first or second, performing better than the other three methods. This is shown in Table 3. Only seven proteins have an allosteric pocket ranked first or second by all four methods. In three cases ExProSE makes a correct prediction for a protein while none of the other methods did.

The performance of the allosteric prediction methods is also compared with the pocket prediction methods LIGSITE^*cs*^ and Fpocket (Le Guilloux et al., 2009) in Table 3. LIGSITE^*cs*^ and Fpocket are effective at finding allosteric sites, both ranking an allosteric pocket first or second for 31 out of 58 proteins, even though they are not designed specifically for allosteric site prediction. This is not too surprising as the holo structures were used as input, so the modulator had a reasonable chance of being in one of the two largest pockets. However, ExProSE is still valuable as it finds smaller, less obvious allosteric pockets. This could be due to the extra structural information used as input. For example, in six cases ExProSE finds sites not ranked in the top 2 by LIGSITE^*cs*^ and in eight cases finds sites not ranked in the top 2 by Fpocket. ExProSE shows the best complementarity to the pocket prediction methods along with STRESS, which makes fewer correct predictions. ExProSE also gives information on how the ensemble may be affected by the modulators, as demonstrated in Figure 4, allowing inspection of the predicted structural and dynamic changes arising from perturbation.

The performance on each protein by each method is shown in Table S2. This is, to our knowledge, the first systematic comparison of multiple allosteric prediction methods. Forty-nine of 58 proteins had an allosteric pocket ranked first or second by at least one of the six methods compared in Table 3. This complementarity indicates the potential for a meta-approach that combines predictions from multiple methods.

### Dynamic Allostery in CAP

Catabolite activator protein (CAP) is a transcriptional activator that exists as a homodimer. Each subunit has a ligand binding domain at the N terminus and a DNA binding domain at the C terminus. Two cyclic AMP (cAMP) molecules bind CAP with negative cooperativity and increase the affinity of the protein for DNA. The negative cooperativity of cAMP binding is a well-studied example of dynamic, or entropic, allostery (Popovych et al., 2006). The binding of one cAMP does not significantly change the structure of the other cAMP binding site, but changes in the dynamics at the other site make binding entropically unfavorable (Popovych et al., 2006, Louet et al., 2015).

**Figure 5:**
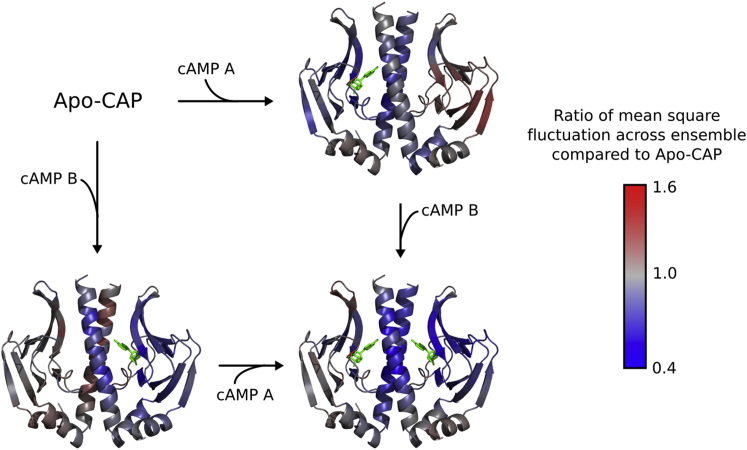
Mean Square Fluctuations across CAP Ensembles Compared with Apo-CAP The four ensembles are generated separately. Apo-CAP has no cAMP. The other ensembles have additional constraints (see Experimental Procedures) representing cAMP bound to chain A, cAMP bound to chain B, or cAMP bound to both chains A and B. The bound cAMP molecules are shown for reference as green sticks. Red regions indicate residues with more flexibility compared with apo-CAP, and blue regions indicate residues with less flexibility compared with apo-CAP.

ExProSE was used to explore the dynamic allostery in CAP. A single structure was used as input (PDB: 1G6N) and four ensembles were generated with perturbations representing no cAMP bound (apo-CAP), cAMP bound to chain A, cAMP bound to chain B, and cAMP bound to both chains A and B. Note that this is the only case in this study whereby a single structure, rather than two, was used as input. The mean square fluctuation across each ensemble was calculated for each residue and gives a measure of the conformational flexibility of the residue across the ensemble. By comparing the mean square fluctuation of the ensembles with one or two cAMP bound to the ensemble of apo-CAP, we can see how the binding of cAMP affects the conformational flexibility of the protein. Figure 5 shows this visually.

On binding cAMP to chain A, the surrounding regions of chain A become more rigid. This is to be expected on ligand binding. However, significant regions of chain B have the same flexibility (gray regions in Figure 5) or are more flexible (red regions) on ligand binding to chain A. The corresponding effect happens on a single cAMP binding to chain B. However, on cAMP binding to both chains, both binding sites become significantly rigid and nearly all regions of the protein are more constrained than in apo-CAP. The ratio of mean square fluctuations as seen in Figure 5 follows the order parameter data and amide exchange rates, which from a previous study are a measure of flexibility in the protein (Popovych et al., 2006). The explanation for the negative cooperativity given in the existing study is that the binding of the second cAMP significantly quenches motions in the protein; this has an associated entropic cost that leads to negative cooperativity between the cAMP sites. The data from ExProSE support this conclusion.

The structural changes on cAMP binding were also measured using ExProSE. The average structures across the ensembles of apo-CAP, and CAP with cAMP bound to chain A, were compared. The RMSD of chain A and chain B between the averages of the ensembles was 0.16 Å and 0.08 Å, respectively. This indicates minor structural rearrangement in chain A due to ligand binding, but almost no change in chain B. This agrees with chemical shift mapping in the existing study (Popovych et al., 2006). These results indicate that ExProSE is able to reproduce dynamic allostery in a model system.

## Discussion

The allosteric prediction methods PARS, STRESS, and AlloPred all use NMA to predict allosteric sites. NMA is computationally inexpensive and hence suitable for high-throughput, automated approaches. However, the assumption of harmonic fluctuations around an energetically minimum structure often makes prediction of conformational changes difficult, particularly for transitions with a low degree of collectivity (Yang et al., 2007). In addition, the choice of which normal modes to use is non-trivial. Larger conformational changes are associated with low-frequency normal modes, but higher-frequency modes are also required to take into account local effects. The focus of NMA on changes in dynamics is also important: while NMA-based methods might be expected to reveal perturbations to vibrations in proteins, there are a variety of other motions that contribute to allostery, such as local unfolding and rigid body movements (Motlagh et al., 2014). By contrast, ExProSE generates native-like protein structures accounting for various interactions (Table 4) that can span large conformational changes. The structure generation process is then perturbed to predict allosteric sites. This has the potential to discover effects not revealed by NMA-based methods while retaining the low computational cost and ease of use. It also provides an ensemble of structures under the influence of the predicted modulator that can be used, for example, in flexible ligand docking. Energy minimization provides a way to improve the stereochemistry of generated structures for use in such approaches.

ExProSE requires two structures for each protein, whereas other methods only require one. It also requires the structures to be different from each other in order to generate structures that span the conformational space. This makes the method unsuitable for use on proteins where only one structure, or highly similar structures, is available. However, many medically important proteins have multiple structures available, including the examples used in this study. In these cases it makes sense to use the additional structural information. The method also was successful at reproducing the allostery in CAP using only one structure as input. For proteins with multiple different conformational states, more than two structures could be used as input to ExProSE to explore further regions of conformational space: the constraint combination procedure can be applied to an arbitrary number of structures.

For many ensemble generation methods, such as MD and tCONCOORD, the choice of parameters has a large effect on the structures produced. The parameter in ExProSE with the largest effect is *W*_*B*_ (see Experimental Procedures), which affects the conformational spread of the ensemble. Without any user input, the auto-parameterization step of ExProSE selects a value that gives an ensemble a wide spread over the conformational space between the two input structures. Once *W*_*B*_ has been selected automatically, an ensemble that spans the correct space is generally produced without any further choice of parameters. This makes the method suitable for high-throughput structure generation across multiple proteins, as users do not need to make any parameter choices themselves. The auto-parameterization procedure can be adjusted to obtain the desired level of structural flexibility using the parameter *F*, which is intuitive in terms of the spread of structures over conformational space (see Experimental Procedures). This provides a way to generate an ensemble with more flexibility if the input structures are similar, as mentioned above.

In this study, LIGSITE^*cs*^ was used to predict pockets for ExProSE. However, it is worth noting that any pocket prediction method that outputs pocket points is compatible with ExProSE without modification. One of the challenges in allosteric site prediction is discovery of transient pockets, i.e., pockets that are only present in some structures of the ensemble. There are currently no general methods that use transient pockets for allosteric site prediction (Boehr et al., 2009), although recent studies have used Markov state models on MD simulations to predict cryptic allosteric sites on multiple proteins (Bowman and Geissler, 2012, Bowman et al., 2015). These studies concluded that cryptic allosteric sites are more ubiquitous than previously thought. ExProSE has the potential to identify transient pockets and predict their ability as allosteric sites. For example, an ensemble could be clustered into a few representative structures, and perturbation at sites on these structures could be used to predict transient allosteric pockets.

ExProSE builds on existing methods by using more structural information as input. It is able to generate ensembles of protein structures that span relevant conformational changes in proteins. This makes it an effective alternative to similar methods and to MD, which is often not feasible for running on timescales long enough to explore large motions of interest without specialist approaches. The perturbation procedure can be applied systematically to predict allosteric sites. In a comparison of multiple allosteric site predictors, ExProSE showed performance similar to and complementary with existing methods. Experimental results in the well-studied CAP were also reproduced by ExProSE. The ability to generate ensembles of protein structures and investigate the response of an ensemble to perturbations should prove useful for both the exploration of individual proteins and the systematic study of the whole PDB. Such methods are required to make sense of the increasing volume of structural data and to understand the crucial importance of dynamics to protein function.

## Experimental Procedures

ExProSE is based on the CONCOORD distance geometry method (de Groot et al., 1997), but has important differences that make it suitable for modeling conformational transitions and ensemble perturbations. These are primarily the use of two input structures instead of one, a different procedure for achieving convergence, the ability to predict the effect of a modulator, and an auto-parameterization procedure. ExProSE is implemented in Julia, a language that combines readable syntax similar to Python with performance approaching statically compiled languages such as C. Use of Julia allows good computational performance at the limiting steps, but also allows compact and easy-to-use code that others can modify. The code, documentation, details of the datasets, and instructions for reproducing the data are freely available under the MIT license as a Julia package at https://github.com/jgreener64/ProteinEnsembles.jl. The code is written in a modular way with associated unit tests and an automated building and testing procedure.

### Distance Constraint Generation

The first step is to obtain a set of distance constraints from a protein structure. Contrary to similar studies (Panjkovich and Daura, 2012, Huang et al., 2013) the smallest biological assembly of the protein is used, rather than only the chain containing the allosteric modulator. Hetero atom records, including the allosteric modulators, are removed. Any existing hydrogens are removed and polar hydrogens are added using an in-house script. Secondary structure assignments, required to obtain additional distance constraints, are obtained using the DSSP software (Touw et al., 2015). As two structures for the same protein are utilized to generate distance constraints, only atoms common to both structures are used. Every atom pair is examined and assigned an interaction type. The criteria for each interaction are the same as in CONCOORD (de Groot et al., 1997) and are shown in Table 4.

Each atom pair is assigned the first interaction for which it fulfills the criterion. If an atom pair is not assigned any of the first 14 specific interactions, it is assigned the generic “all other pairs” interaction type. Lower and upper distance constraints *l*_*ij*_ and *u*_*ij*_ are generated for each atom pair *ij* based on the interatomic distance *d*_*ij*_, the constraint tolerance for the interaction *t*_*ij*_ and a tolerance weighting factor *W*_*B*_ that is between 0.0 and 1.0:

*l*_*ij*_ = *d*_*ij*_ − *W*_*B*_*t*_*ij*_, *u*_*ij*_ = *d*_*ij*_ + *W*_*B*_*t*_*ij*_

The selection of *W*_*B*_ is described below. For example, two atoms 1.54 Å apart and in a covalent bond with *W*_*B*_ equal to 0.5 would have a lower distance constraint of 1.53 Å and an upper distance constraint of 1.55 Å, as the constraint tolerance multiplied by *W*_*B*_ is 0.01 Å. This process yields a set of distance constraints for each crystal structure of a protein.

The distance constraints generated from the two structures for the same protein are combined to get a set of combined constraints. The constraints are combined in such a way that the new constraints for a given atom pair cover the distance of both the individual constraints for that pair. For example, if two atoms have a lower and upper distance constraint of 6.0 Å and 7.0 Å in structure 1 and 6.5 Å and 7.5 Å in structure 2, then the new constraints will be 6.0 Å and 7.5 Å.

It is undesirable to retain all the “all other pairs” interactions (type 15 in Table 4) as they vastly outnumber the specific interactions (types 1–14). Specific interactions scale with the atom number *N*_*A*_ whereas other pairs scale as $N_{A}^{2}$. Hence only a fraction of the other pairs are retained as distance constraints. The probability of retaining an other pair is chosen so that the final number of other pairs is roughly 20*N*_*A*_, the value used by studies utilizing CONCOORD (de Groot et al., 1999).

*W*_*B*_ is chosen for each protein in the apo/holo and allosteric datasets by a process of auto-parameterization. *W*_*B*_ equal to 0.0 usually results in a narrow range of structures that are midway between the two input structures. By contrast, *W*_*B*_ equal to 1.0 usually results in structures that cover a wide conformational space beyond the input structures. A measure for the conformational spread of the ensemble was developed. This measure *F* is the fraction of structures *S* in the ensemble for which *TM*(*S*,*A*) > *TM*(*B*,*A*) and *TM*(*S*,*B*) > *TM*(*A*,*B*) where *TM*(*X*,*Y*) is the TM score between model *X* and reference *Y*, and *A* and *B* are the two input crystal structures. The TM score is a measure of similarity between two protein structures. *F* therefore gives the proportion of structures that are closer to both input structures than the input structures are to each other. *F* equal to 0.9 indicates an ensemble that effectively covers the conformational space of the input structures. Ensembles of 50 structures are generated with *W*_*B*_ starting at 1.0 and decreasing in steps of 0.1. When the ensemble generated has an *F* value of at least 0.9, that *W*_*B*_ is chosen. For the specific examples T4-lysozyme and CDK2, *W*_*B*_ is equal to 0.2 and 0.3, respectively. It should be noted that the above auto-parameterization procedure to select *W*_*B*_ is implemented automatically and requires no input by the user. For CAP only one input structure is used, so *W*_*B*_ is selected manually as 0.4. This value allows flexibility in the ensemble while giving good-quality structures.

### Protein Structure Generation

Once the distance constraints have been generated, an iterative process is used to generate structures that satisfy the constraints. Stochastic proximity embedding (SPE) (Agrafiotis et al., 2013) was selected, as it has been shown to converge effectively and scales well with system size. This procedure provides better convergence than the CONCOORD procedure of moving atoms to a random distance within the distance constraints. The pseudocode for the SPE algorithm, rephrased from an existing review (Agrafiotis et al., 2013), is shown in Algorithm S1. The distance constraints do not include favorability for a particular chirality, so coordinates produced from SPE are examined and structures with the incorrect chirality are reversed by mirroring all coordinates in the *xy* plane.

Once a set of coordinates has been generated, an SPE error score can be calculated that measures how well the distance constraints are satisfied (Agrafiotis et al., 2013). This score is calculated as shown in Algorithm S2. Structures with a high error score tend to have more violations of allowed stereochemistry, which is to be expected as there are more violations of allowed constraints. More structures are generated than required and those with the highest scores are discarded to account for this. The ratio is set to be 1.5. So if the final ensemble had 200 structures, initially 300 are generated, and the 100 with the highest error score are discarded. This was found during development to generally produce ensembles of structures with acceptable stereochemical quality.

The number of iterations per atom, the product of the number of cycles *C* and the number of steps *S* from Algorithm S1, is taken as 60,000. This was chosen because the SPE error score did not generally decrease for iterations beyond this. The ratio of *S* to *C* is taken as 50:1, as in practice any value of *S* > *C* will give similar results (Agrafiotis et al., 2013). The reduction in learning rate over the course of the minimization makes this process similar to simulated annealing. Initially large movements through the conformational space allow the correct region to be found. The movements are dampened over time to allow the system to converge to a solution. This procedure is carried out separately multiple times to obtain an ensemble.

### Ensemble Analysis

Ensembles of structures produced are iteratively aligned following the procedure described in the methodology of a previous study (Bakan and Bahar, 2009). This aligns an ensemble without the use of a reference structure. The average structure of the ensemble is taken as the centroid of the coordinates across the ensemble following this superimposition.

PCA is carried out on the generated ensemble. The coordinates across the ensemble are compared with the average coordinates, and a set of orthogonal motions are found that describe the variation in the ensemble. The covariance matrix *C*_*ij*_ is a matrix where *i* and *j* represent the indices of the 3*N*_*C*_ atomic coordinates of the *N*_*C*_ Cα atoms. *C*_*ij*_ is calculated as

*C*_*ij*_ = 〈(*x*_*i*_ − 〈*x*_*i*_〉)⋅(*x*_*j*_ − 〈*x*_*j*_〉)〉,

where the averages in angle brackets are over the ensemble and *x* represents the atomic coordinates. *C* is then diagonalized to yield the PCs.

### Modulator Constraint Generation

Additional distance constraints representing the modulator need to be generated to predict how a modulator binding to the protein affects the distribution of structures in conformational space. Potential binding sites are predicted using LIGSITE^*cs*^ (Huang and Schroeder, 2006), which is a development of the original LIGSITE algorithm (Hendlich et al., 1997). Additional constraints are generated based on pocket points predicted by LIGSITE^*cs*^. A total of 120 points are chosen randomly to keep the number of additional points the same for pockets of different sizes. If fewer than 120 points are predicted by LIGSITE^*cs*^, points are resampled. Using 120 points was found for CDK2 and CAP to add enough constraints to potentially alter the distribution of the ensemble and observe an effect, but not so many that invalid structures are produced. Changing this parameter changes the strength of the perturbations but does not generally change the ranking of pockets by RMSD (see below). For CAP a different procedure was used, as the location of the bound cAMP molecules is known from the crystal structure. In this case 120 fake points are added at 1.2-Å gaps in a ball around the location of the C1′ atom in cAMP, while the cAMP molecules are themselves omitted from the simulation. Selected points have distance constraints of tolerance of 0.1 Å with all protein atoms within 7 Å. Addition of the new distance constraints leads to ensembles that may differ significantly from the unperturbed ensemble.

In the allosteric prediction procedure, ensembles are generated with additional constraints (termed “perturbation”) at selected pockets in turn, then compared with the original “unperturbed” ensemble. Each pocket greater than a size cutoff of 13 Å^3^ is selected, up to a maximum of eight pockets per protein. Below this size a small-molecule modulator is unlikely to have enough space to bind. Eight pockets gives a reasonable sampling of the surface of a protein and generally includes all sizable pockets. The Cα RMSD between the average structure in the unperturbed ensemble and the average structure in the perturbed ensemble is used to compare ensembles. This RMSD is used to rank the perturbed pockets in terms of their predicted allosteric nature (largest to smallest RMSD). A pocket is considered allosteric for validation purposes if the pocket center is within 6 Å of at least one atom of the modulator defined as the allosteric modulator in the ASD. This is similar to previous studies (Panjkovich and Daura, 2012).

### Apo/Holo Dataset

Of the 25 proteins used in a prior study (Atilgan et al., 2010), the 12 with apo/holo all-atom RMSD greater than 2 Å are selected in order to focus on larger conformational changes.

### Allosteric Dataset

All 150 proteins in the ASD (Shen et al., 2016) with apo and holo structures available in the PDB are examined. Fifty-eight proteins with apo and holo structures are selected using the following criteria: (1) apo/holo all-atom RMSD greater than 0.25 Å, (2) TM score greater than 0.5, and (3) no more than two chains and 1,000 residues in the smallest biological assembly. Proteins are also clustered by sequence identity at a threshold of 30%, with representatives being the proteins with the highest apo/holo RMSD, to remove similar proteins.

### Method Comparison

#### Ensemble Generation

tCONCOORD (Seeliger et al., 2007) is run with default parameters. NMSim is run via the NMSim web server (Kruger et al., 2012) with the default parameters for large-scale motions. This produces five trajectories of 500 structures. Every tenth structure is taken from each trajectory to yield representative ensembles of 250 structures. Alternative parameters for tCONCOORD and NMSim are used to generate the results in Figure S1, and these are described in the figure.

#### Molecular Dynamics

All MD runs are carried out using the GROMACS package (Abraham et al., 2015). Energy minimization to improve the stereochemistry of T4-lysozyme structures is conducted using a steepest descent energy minimization of 5,000 steps in a vacuum and the OPLS-AA force field. MD runs of T4-lysozyme are conducted using periodic boundary conditions, SPC water, charge-neutralizing counter ions, the OPLS-AA force field, and a 2-fs time step. An initial energy minimization is followed by a constant temperature and volume equilibration for 100 ps, then a constant pressure and temperature equilibration for 100 ps. MD is run for 50 ns. PLUMED (Tribello et al., 2014) with GROMACS is used to carry out targeted MD. Cα RMSD to the target structure is used as a collective variable with a *κ* value starting at 0 kJ mol^−1^ Å^−2^ and increasing linearly to 1,000 kJ mol^−1^ Å^−2^ over 10 ps, and remaining at this value for the rest of the run.

#### Allosteric Site Prediction

LIGSITE^*cs*^ (Huang and Schroeder, 2006) and Fpocket (Le Guilloux et al., 2009) are run with default parameters. The procedure for determining whether an Fpocket pocket is allosteric is as follows: the average of the locations of the vertices in the pocket is taken as the pocket center, and the pocket is considered allosteric if this center was within 6 Å of at least one atom of the modulator defined as the allosteric modulator in the ASD. This is consistent with the criterion for determining LIGSITE^*cs*^ allosteric pockets defined previously. PARS results are obtained by using the PARS web server (Panjkovich and Daura, 2014). PARS uses LIGSITE^*cs*^, so the same criterion as LIGSITE^*cs*^ is used to determine allosteric pockets. AlloPred is run using the offline version (Greener and Sternberg, 2015) and default parameters. The active-site residues are retrieved from the Catalytic Site Atlas (CSA) (Furnham et al., 2014), or from literature inspection when not available in the CSA. AlloPred uses Fpocket, so the same criterion as Fpocket is used to determine allosteric pockets. STRESS (Clarke et al., 2016) is run offline using the source code. Since the output of STRESS is pocket residues, a pocket is called as allosteric if there is at least one modulator atom within 3 Å of any atom in the given residues of the pocket. This represents the modulator being close to part of the predicted pocket. This value of 3 Å is less than the value of 6 Å used previously, as there are many residues which the modulator can be close to, rather than a single pocket center.

#### Computation Time

ExProSE generates 250 structures in ∼20 min for T4-lysozyme on a 3.1-GHz Intel Core i7 processor. For tCONCOORD the time is ∼10 min. NMSim is run via the NMSim web server and takes ∼5 hr. MD and targeted MD use considerably more resources, with a 50-ns run taking ∼60 hr on 16 cores (2.3-GHz Intel Xeon CPU E5-2698) or ∼20 days on the single processor above.

## Author Contributions

J.G.G., I.F., and M.J.E.S. conceived and designed the study. J.G.G. wrote the software, performed the computational work, analyzed the data, and prepared the manuscript. All authors edited the manuscript.

## Figures and Tables

**Table 1:** Comparison of Ensemble Generation Methods

					tCONCOORD from Apo		tCONCOORD from Holo		NMSim from Apo		NMSim from Holo	
					Lowest RMSD from 250 Generated Structures to Apo/Holo Crystal (Å)							
Protein Name	Apo PDB	Holo PDB	RMSD (Å)	N	Apo	Holo	Apo	Holo	Apo	Holo	Apo	Holo
OxyR transcription factor	1I6A	1I69	2.44	206	1.18	2.69	2.66	1.12	1.04	2.61	2.51	0.72
Ferric binding protein	1D9V	1MRP	2.68	309	1.22	1.81	1.88	1.41	0.62	2.07	2.31	0.71
Aspartate receptor	1LIH	2LIG	2.77	157	1.16	2.73	2.94	1.48	0.94	2.45	2.65	0.80
HIV-1 reverse transcriptase	2HMI	3HVT	3.81	555	2.49	4.11	4.66	3.44	0.64	3.28	3.14	0.78
Maltose binding protein	1OMP	3MBP	3.88	370	0.97	2.62	2.66	0.89	0.71	2.35	2.39	0.57
Small G protein Arf6	1E0S	2J5X	4.44	164	0.99	4.18	4.23	0.96	0.66	4.00	4.23	0.86
Immunoglobulin	1MCP	4FAB	5.95	214	1.65	3.60	3.80	1.51	0.62	5.35	3.63	0.79
Myosin	1VOM	2AKA	6.23	730	2.60	5.11	5.63	2.38	0.73	5.53	5.77	0.63
Adenylate kinase	4AKE	1AKE	7.19	214	1.70	4.88	6.00	1.18	0.58	6.16	6.09	0.74
Serpin	1PSI	7API	8.96	372	1.20	8.71	8.93	1.51	0.71	8.22	8.97	0.97
GroEL	1AON	1OEL	12.6	524	3.01	9.72	9.61	2.45	0.87	10.8	10.1	0.48
Topoisomerase II	1BGW	1BJT	18.8	664	3.36	17.5	17.0	3.34	0.81	18.0	17.3	0.65
Median across all proteins					1.44	4.15	4.45	1.50	0.71	4.68	3.93	0.73

The columns Apo PDB and Holo PDB refer to the PDB IDs of the apo and holo structures used. RMSD is the all-atom RMSD (Å) between the apo and holo structures. The rows are ordered by increasing RMSD. N is the number of residues in common between the apo and holo chains used. The values on the right are the lowest RMSD in Å of the structures in an ensemble produced using the method and input indicated, to the crystal structure indicated. A low value indicates that the ensemble sampled a structure close to the crystal structure. The median of the lowest RMSDs for each method/input combination is also given.

**Table 2:** Ability of ExProSE Ensembles to Reach Apo and Holo Structures

					Lowest RMSD from 250 Generated ExProSE Structures to Apo/Holo Crystal (Å)	
Protein Name	Apo PDB	Holo PDB	RMSD (Å)	N	Apo	Holo
OxyR transcription factor	1I6A	1I69	2.44	206	1.02	1.16
Ferric binding protein	1D9V	1MRP	2.68	309	0.90	1.08
Aspartate receptor	1LIH	2LIG	2.77	157	1.25	0.88
HIV-1 reverse transcriptase	2HMI	3HVT	3.81	555	1.84	1.45
Maltose binding protein	1OMP	3MBP	3.88	370	0.85	1.50
Small G protein Arf6	1E0S	2J5X	4.44	164	1.70	1.88
Immunoglobulin	1MCP	4FAB	5.95	214	3.90	5.33
Myosin	1VOM	2AKA	6.23	730	2.38	1.89
Adenylate kinase	4AKE	1AKE	7.19	214	3.15	1.98
Serpin	1PSI	7API	8.96	372	1.08	1.01
GroEL	1AON	1OEL	12.6	524	3.13	3.70
Topoisomerase II	1BGW	1BJT	18.8	664	3.54	5.10
Median across all proteins					1.77	1.69

The columns Apo PDB, Holo PDB, RMSD, and N are the same as in Table 1. The values on the right are the lowest RMSD (Å) of the structures in an ExProSE ensemble to the crystal structure indicated. A low value indicates that the ensemble sampled a structure close to the crystal structure. The median of the lowest RMSDs is also given.

**Table 3:** Performance of Allosteric Site Prediction Methods on a Dataset of 58 Known Allosteric Proteins

Method	Correct in Top 2 (Out of 58)	Unique from LIGSITE^*cs*^	Unique from Fpocket
ExProSE	27	6/27	8/27
PARS	25	3/25	7/25
STRESS	18	6/18	8/18
AlloPred	26	5/26	1/26
LIGSITE^*cs*^	31	–	8/31
Fpocket	31	8/31	–

Correct in Top 2 is the number of proteins for which the method successfully ranked an allosteric pocket first or second. The definition of an allosteric pocket is given in Experimental Procedures. The number of correct predictions by each method that are unique from the correct predictions of LIGSITE^*cs*^ and Fpocket is also shown. STRESS could not run on four proteins as they were too small. See also Table S2.

**Table 4:** Interaction Types between Atom Pairs

Number	Interaction Name	Constraint Tolerance (Å)	Definition
1	covalent bond	0.02	pairs that are covalently bonded
2	bond angle	0.05	pairs where both atoms are covalently bonded to the same atom
3	ring	0.1	pairs that are part of ring systems
4	double bond 1–4	0.1	1–4 dihedral angle restricted pairs in side chain double bonds (found in Asn, Gln, and Arg)
5	omega 1–4	0.1	1–4 pairs constrained by the rigid ω dihedral angle
6	tight phi/psi 1–4	0.2	1–4 pairs constrained by the φ/ψ dihedral angle where one residue is a proline or both residues are in the same helix/strand
7	loose phi/psi 1–4	0.4	1–4 pairs constrained by the φ/ψ dihedral angle where one residue is a glycine or both residues are in a loop region
8	other phi/psi 1–4	0.3	1–4 pairs constrained by the φ/ψ dihedral angle that do not fall into the above two categories
9	other 1–4	0.4	other 1–4 dihedral angle restricted pairs that do not fall into the above categories
10	secondary structure	0.5	pairs of backbone atoms that are in the same helix/strand and are not more than 4 residues apart
11	salt bridge	0.75	pairs from oppositely charged groups in close proximity (less than 4 Å apart)
12	hydrogen bond	0.5	pairs that are part of a hydrogen bond; donor-acceptor distance is no more than 3.5 Å, hydrogen-acceptor distance is no more than 2.5 Å, and the donor-hydrogen-acceptor angle is at least 90°
13	tight hydrophobic	0.5	pairs where the interatomic distance is less than the sum of the van der Waals radii of the atoms plus 0.5 Å; only C and H atoms are counted
14	loose hydrophobic	1.0	pairs where the interatomic distance is less than the sum of the van der Waals radii of the atoms plus 1.0 Å; only C and H atoms are counted
15	all other pairs	5.0	pairs that do not fall into any of the above categories

These are the same as in CONCOORD (de Groot et al., 1997). The constraint tolerance values are used to generate lower and upper distance constraints between atoms.

## Supplemental Information

Document S1. Figure S1, Tables S1 and S2, and Algorithms S1 and S2

Document S2. Article plus Supplemental Information
