# Supplementary material for: Predicting Protein Dynamics and Allostery Using Multi-Protein Atomic Distance Constraints
Source: Structure. 2017 Mar 7;25(3):546–58. doi: 10.1016/j.str.2017.01.008 (PMC5343748; doi:10.1016/j.str.2017.01.008)
Supplement: Document S1. Figure S1, Tables S1 and S2, and Algorithms S1 and S2 [file mmc1.pdf]

Structure, Volume *25*

Supplemental Information

Predicting Protein Dynamics and Allostery Using Multi-Protein Atomic Distance Constraints

Joe G. Greener, Ioannis Filippis, and Michael J.E. Sternberg

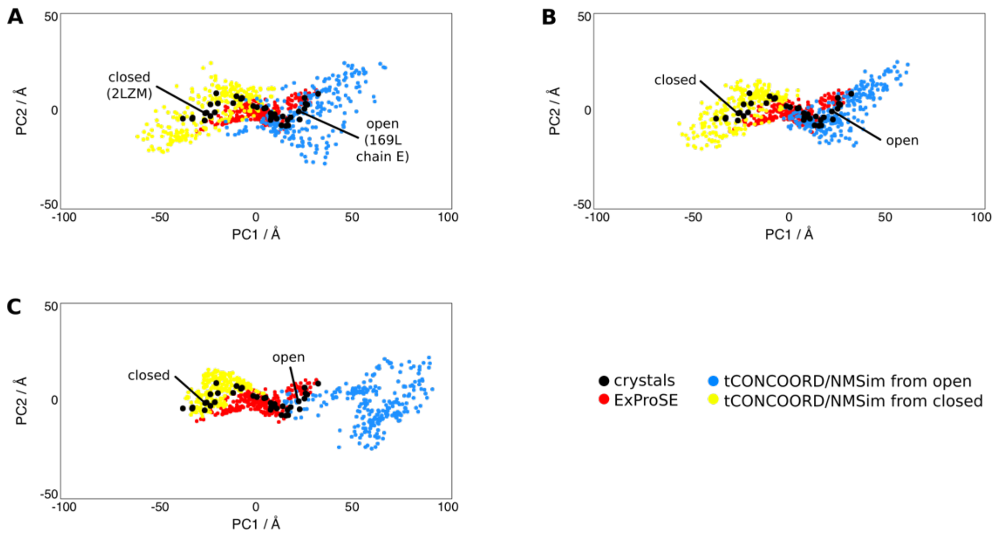

Figure S1. Related to Figure 1 Ensemble generation for T4-lysozyme with different parameters. Projections of tCONCOORD/NMSim ensembles from the open (blue dots) and closed (yellow dots) structures onto the PCA of the crystal structures are shown. Similar to Figure 1, in each graph the projections of the crystals (black dots) and projections from the ensembles generated with ExProSE (red dots) are also shown. (**A**) tCONCOORD ensembles with the upper bound for long range constraints set to 1.3 Å (default 2.0 Å). (**B**) tCONCOORD ensembles with the upper bound for long range constraints set to 1.3 Å and close pairs not used as constraints. (**C**) NMSim ensembles using the default parameters for small scale motions.

Method	Structure(s) used	Median overall G-factor before energy minimisation	Median overall G-factor after energy minimisation
ExProSE	open and closed	-0.58	-0.26
tCONCOORD	open	-2.23	-0.31
tCONCOORD	closed	-2.09	-0.27
NMSim	open	-0.50	-0.31
NMSim	closed	-0.45	-0.29
targeted MD	starting open, targeting closed	-0.56	-0.17
targeted MD	starting closed, targeting open	-0.57	-0.20

Table S1. Related to Figure 1 Improvement in stereochemical quality on energy minimisation. The structures in each ensemble were analysed with PROCHECK and the median overall G-factor across the ensemble was noted. The median of the overall G-factor of each structure after energy minimisation was also recorded.

Protein name	Apo PDB	Holo PDB	Apo chains	Holo chains	*N*	ExProSE	PARS	STRESS	AlloPred	LIGSITE^{cs}	Fpocket
Pyruvate kinase	1A3X	1A3W	AB	AB	955						
Antithrombin-III	1ANT	3KCG	I	I	399						
HIV-1 integrase	1BIZ	4CHO	AB	AB	276						
Chorismate mutase	1CSM	2CSM	AB	2 x A	490						
Plasminogen activator inhibitor 1	1DB2	4AQH	A	A	377						
HTH-type transcriptional repressor purR	1DBQ	1JH9	AB	2 x A	550						
Ribose-phosphate pyrophosphokinase	1DKR	1DKU	AB	AB	588						
Fatty acid metabolism regulator protein	1E2X	1H9G	2 x A	2 x A	444						
Androgen receptor	1E3G	4K7A	A	A	242						
Herpesvirus protease	1FL1	4P3H	A	A	153						
Glutamate receptor 2	1FTO	3ILT	A	B	257						
Annexin A5	1HVG	1HAK	A	A	313						
Neurolysin, mitochondrial	1I1I	4FXY	P	P	664						
Cell division control protein 4	1NEX	3MKS	AB	CD	572						
Phospho-2-dehydro-3-deoxyheptonate aldolase	1OFP	1OFR	AB	GH	628						
Organophosphorus hydrolase	1PTA	1QW7	2 x A	AB	636						
Ribonucleotide reductase	1RLR	3UUS	A	A	727						
Cytochrome P450 3A4	1W0E	1W0F	A	A	452						
Acetyl-CoA carboxylase	1W93	1W96	A	A	549						
Hypothetical biotin--[acetyl-CoA-carboxylase] ligase	1WQ7	2DVE	AB	AB	456						
Putative uncharacterized protein PH0207	1X0M	3ATH	A	A	403						
Integrin alpha-L	1ZON	1RD4	A	A	181						
Pyruvate dehydrogenase kinase isoform 2	2BTZ	2BU2	2 x A	2 x A	708						
Farnesyl pyrophosphate synthase	2F7M	3N45	2 x F	2 x F	682						
Fructose-1,6-bisphosphatase	2FBP	1Q9D	AB	AB	630						
Protein arginine N-methyltransferase 3	2FYT	3SMQ	A	A	299						
Glycogen phosphorylase	2GPN	1PYG	A	A	787						
Glutamate racemase	2JFX	4B1F	AB	AB	498						
Myosin-2 heavy chain	2JJ9	2JHR	A	A	692						
Ubiquitin-conjugating enzyme E2 R1	2OB4	3RZ3	A	A	153						
Cytosolic purine 5'-nucleotidase	2XCX	2JC9	2 x A	2 x A	916						
cAMP receptor protein	3D0S	3I54	AB	AB	422						
Endothelial PAS domain-containing protein 1	3F1P	3H82	AB	AB	222						
Acetylcholinesterase	3GEL	2J3Q	A	A	527						
NAD-dependent deacetylase sirtuin-3, mitochondrial	3GLU	4C7B	AB	AB	261						
FimX	3HV9	3HV8	A	A	242						
Glucokinase	3IDH	4ISE	A	A	419						
Glutamate receptor ionotropic, NMDA 2B	3JPW	3QEL	A	B	349						
Global nitrogen regulator	3LA7	3LA3	AB	AB	382						
Genome polyprotein	3MWV	4JTZ	A	A	559						
Beta-lactamase SHV-1	3N4I	1VM1	A	A	265						
DNA double-strand break repair Rad50 ATPase	3QG5	3THO	A	A	349						
N-acetylglutamate kinase / N-acetylglutamate synthase	3S7Y	4KZT	AX	AX	862						
Leucine transporter	3TU0	2QEI	A	A	509						
6-phosphofructokinase isozyme 2	3UMP	3CQD	AB	AB	612						
Kinesin-like protein KIF11	4A28	4BXN	A	A	330						
Eukaryotic translation initiation factor 4E	4BEA	4TQC	A	A	174						
Penicillin binding protein 2 prime	4BL2	3ZG0	A	A	636						
CAMP-dependent protein kinase	4DFY	4DFX	A	E	311						
Casein kinase II	4DGL	3H30	C	A	333						
Mitogen-activated protein kinase 14	4E5B	3NNX	A	A	321						
PelD	4ETX	4ETZ	A	A	285						
Caspase 7	4FDL	4FEA	AB	AB	365						
Glucose-1-phosphate thymidylyltransferase	4HO0	4HO9	A	A	285						
GTPase Kras	4LPK	4LUC	A	A	156						
CRP transcriptional dual regulator	4N9H	4N9I	AB	AB	402						
2-C-methyl-D-erythritol 4-phosphate cytidylyltransferase	4NAI	2YC3	2 x A	2 x A	428						
Adenylate cyclase type 10	4OYW	4USW	A	A	458						
**Found in top 2 predicted – out of 58 (54 for STRESS)**						27	25	18	26	31	31

Table S2. Related to Table 3 Performance of allosteric site prediction methods on a dataset of 58 known allosteric proteins. Apo PDB and Holo PDB refer to the PDB IDs of the apo and holo structures used. Apo chains and Holo chains are the chains utilised from the apo and holo structures. 2 x A means chain A is duplicated as part of a biological assembly. *N* is the number of residues in common between the apo and holo chains used. A green square indicates that the method ranked an allosteric pocket first or second for that protein. The definition for an allosteric pocket is given in the experimental procedures. A red square indicates that the method failed to rank an allosteric pocket first or second. STRESS could not run on 4 proteins as they were too small - this is indicated by a yellow square.

Define lower and upper distance constraints l_{ij} and u_{ij} for atom pairs i and j
Define an initial learning rate $\lambda_d = 1.0$
Randomise atomic coordinates x_i within a cube of 100 Å
for C cycles **do**
    for S steps **do**
        Randomly select a pair of atoms i and j for which a constraint exists
        Compute the distance $d_{ij} = \|x_i - x_j\|$
        if $d_{ij} < l_{ij}$ or $d_{ij} > u_{ij}$ **then**
            Update the coordinates x_i and x_j by

$$x_i = x_i + \frac{\lambda_d}{2} \frac{t_{ij} - d_{ij}}{d_{ij}} (x_i - x_j)$$
$$x_j = x_j + \frac{\lambda_d}{2} \frac{t_{ij} - d_{ij}}{d_{ij}} (x_j - x_i)$$

            where t_{ij} is the nearest constraint to d_{ij}
        end if
    end for
    Decrease the learning rate λ_d by $1/C$
end for

Algorithm S1. Related to the Experimental Procedures The stochastic proximity embedding (SPE) algorithm used to move atoms to satisfy distance constraints. Rephrased from Agrafiotis et al. 2013.

Set score $s = 0$
for each atom pair i, j with a distance constraint **do**
    if $d_{ij} < l_{ij}$ or $d_{ij} > u_{ij}$ **then**
        Increase s by

$$\frac{(d_{ij} - t_{ij})^2}{\max(u_{ij} - l_{ij}, 0.001)}$$

        where t_{ij} is the nearest constraint to d_{ij}
    end if
end for

Algorithm S2. Related to the Experimental Procedures The scoring algorithm to calculate the SPE error score. Rephrased from Agrafiotis et al. 2013.
